# Exploring the feasibility, acceptability and preliminary efficacy of a Home-Based stretching program for adults with fibromyalgia: a prospective Pre-Post feasibility study

**DOI:** 10.1007/s00296-025-05921-4

**Published:** 2025-08-11

**Authors:** Morten Pallisgaard Støve, Louise Landbo Larsen, Stig Peter Magnusson, Janus Laust Thomsen, Allan Riis

**Affiliations:** 1 Department of Physiotherapy, University College of Northern Denmark (UCN), Selma Lagerløfs Vej 2, 9220 Aalborg East, Denmark; 2 Center for General Practice, Aalborg University, Selma Lagerløfs Vej 249, 9260 Gistrup, Denmark; 3 Institute of Sports Medicine Copenhagen & Department of Occupational and Physical Therapy, Bispebjerg Hospital, Building 8, 1st Floor, Bispebjerg bakke 23, 2400 Copenhagen NV, Denmark; 4 Center of Healthy Aging, University of Copenhagen, Blegdamsvej 3B, 2200 Copenhagen N, Denmark

**Keywords:** Feasibility studies, Fibromyalgia, Muscle stretching exercises, Quality of life, Rehabilitation

## Abstract

People with fibromyalgia face physical and cognitive impairments and are often intolerant to physical activity, making adherence to physical therapy a significant challenge. This prospective pre-post feasibility study aimed to explore the feasibility and acceptability of a six-week home-based stretching intervention for people with fibromyalgia. People aged 18–55 years diagnosed with fibromyalgia were eligible for participation. Participants were offered a home-based stretching intervention with weekly follow-up via a mHealth app. Semi-structured online focus group interviews were conducted to gain insight into the participants’ experience and acceptance of the intervention. Quantitative assessments included the Revised Fibromyalgia Impact Questionnaire, the SF-36, pressure pain thresholds, range of motion, and self-reported adherence. 12 females were recruited over 43 days. One participant withdrew from the study due to illness. The adherence rate was high. Four major themes emerged from the interviews: (1) Factors motivating participation, (2) The advantages of exercising at home, (3) Influence of weekly communication and (4) Potential areas for improvement. Qualitative findings suggest that the intervention was well tolerated and easily implemented in daily life. There were clinically relevant improvements in health-related quality of life, physical health, and mental health scores. A six-week home-based stretching program, supervised via an mHealth application, proved feasible and acceptable for individuals with FM and showed promising clinical outcomes. Based on insights from this feasibility study, an RCT is planned to evaluate whether the program of home-based stretching exercises provides greater benefits than usual care alone in enhancing quality of life and functional outcomes in patients with FM. *Trial registration number*: NCT06176053. Date of registration: 12/08/2023.

## Introduction

With a prevalence between 2 and 8%, depending on the diagnostic criteria used [1]. Fibromyalgia (FM) is considered the second most common rheumatic disorder behind osteoarthritis [2]. FM is a complicated pain syndrome characterised by chronic widespread pain and generalised hyperalgesia [3]. In addition to pain, other symptoms include fatigue, insomnia, and muscle weakness [4]. Patients with FM are sensitive to physical activity and often suffer from systemic symptoms such as cognitive dysfunction, sleep disturbances, muscle tightness and emotional distress, such as anxiety and depression [5]. FM is also associated with poor quality of life, increased prevalence of depression and diabetes, poor self-rated health, increased illness worry and extensive utilisation of medical services [6–8]. Although the pathophysiological causal factors of FM are not yet well known, sensitisation is considered the primary driver of FM [4, 5].

The current treatment of FM is based on symptom management and includes pharmacological and non-pharmacological interventions [9]. Pharmacological treatment alone is often inadequate. Hence, multidisciplinary treatment programs are advocated for patients with FM [4, 9]. Physical exercises are recommended, given their positive effect on pain, physical function and well-being, and low cost [9]. Current evidence indicates that patients with fibromyalgia who demonstrate higher adherence to treatment tend to report better quality of life [10]. However, adherence to exercise can be challenging, and patients often refrain from physical activity for fear of worsening symptoms [11]. Therefore, a concern in treating FM patients is low adherence to long-term physical exercise programs. Recent evidence also suggests that individuals with fibromyalgia may discontinue physiotherapy or exercise over time, as financial constraints can make sustained participation unfeasible [12]. Therefore, it is important to explore alternative low-cost treatment options, such as home-based exercise programs. However, given that challenges with adherence have been previously reported for both face-to-face and home-based exercise programs [13], it is important to gain a deeper understanding of participants’ acceptance of the interventions and adherence behaviours before initiating clinical trials [14]. Various strategies have been suggested to improve treatment adherence, with mHealth applications, offering remote monitoring and enhanced patient-provider communication, emerging as particularly promising [15]. However, their impact on adherence to home-based exercise programs in individuals with FM remains largely unexplored, though they may hold promising potential.

Current evidence indicates that low-dose exercise modalities are beneficial for individuals with fibromyalgia and are generally well tolerated, with minimal side effects [16]. Furthermore, recent systematic reviews of stretching interventions for individuals with FM show potential improvements in pain, health-related quality of life and physical and mental functioning in people with FM but highlight the need for further high-quality trials [14, 17]. Accordingly, a gap remains in the non-pharmacological treatment of fibromyalgia. Therefore, further research is needed to assess the feasibility, acceptability, and preliminary efficacy of home-based stretching exercises [14, 18] that may promise to improve the impact of FM symptoms without the risk of initially exacerbating them.

In preparation for a full-scale randomised controlled trial, this study aimed to investigate the feasibility and acceptability of a six-week home-based stretching programme in people with fibromyalgia. We used a combination of qualitative and quantitative research, including online focus-group interviews focusing on acceptability and feasibility, which are crucial for identifying potential challenges with recruitment, adherence and retention of the intervention.

## Methods

This prospective feasibility study used a pre-post design [19] supplemented with online focus group patient interviews. The study was designed to evaluate the feasibility, acceptability and preliminary efficacy of a six-week home-based exercise intervention among people living with FM. The study adopted a hermeneutic method [20]. The trial was approved by the North Denmark Region Committee on Health Research Ethics (N-20230056), reported to the Danish Data Protection Agency and registered at ClinicalTrials.gov (12/08/2023) (Trial registration number NCT06176053). A protocol for the randomised controlled trial has been published [21]. Participants provided written informed consent before participation. Enrolment began on February 13th, 2024 and ended on May 30th, 2024.

### Participants

People aged 18–55 years diagnosed with FM in accordance with the ACR diagnostic criteria [22] were eligible for participation. Exclusion criteria included (1) non-controlled systemic disorders (such as hypertension, diabetes and coronary insufficiency), (2) neurological conditions that impair alertness or comprehension, (3) musculoskeletal conditions that could compromise assessments (such as nerve root compression or knee joint inflammation), (4) relevant joint disorders (such as severe arthritis, arthroplasty of the hip or knee, and rheumatoid arthritis), (5) and recent changes in therapy for FM (i.e., within four weeks of baseline).

Participants were recruited through posts from the Danish Fibromyalgia & Pain Association in the North Denmark Region and social media. Patients interested in the study contacted MPS by phone or e-mail. After obtaining verbal consent, MPS administered initial study information and eligibility screening by telephone.

### Intervention

**Fig. 1:**
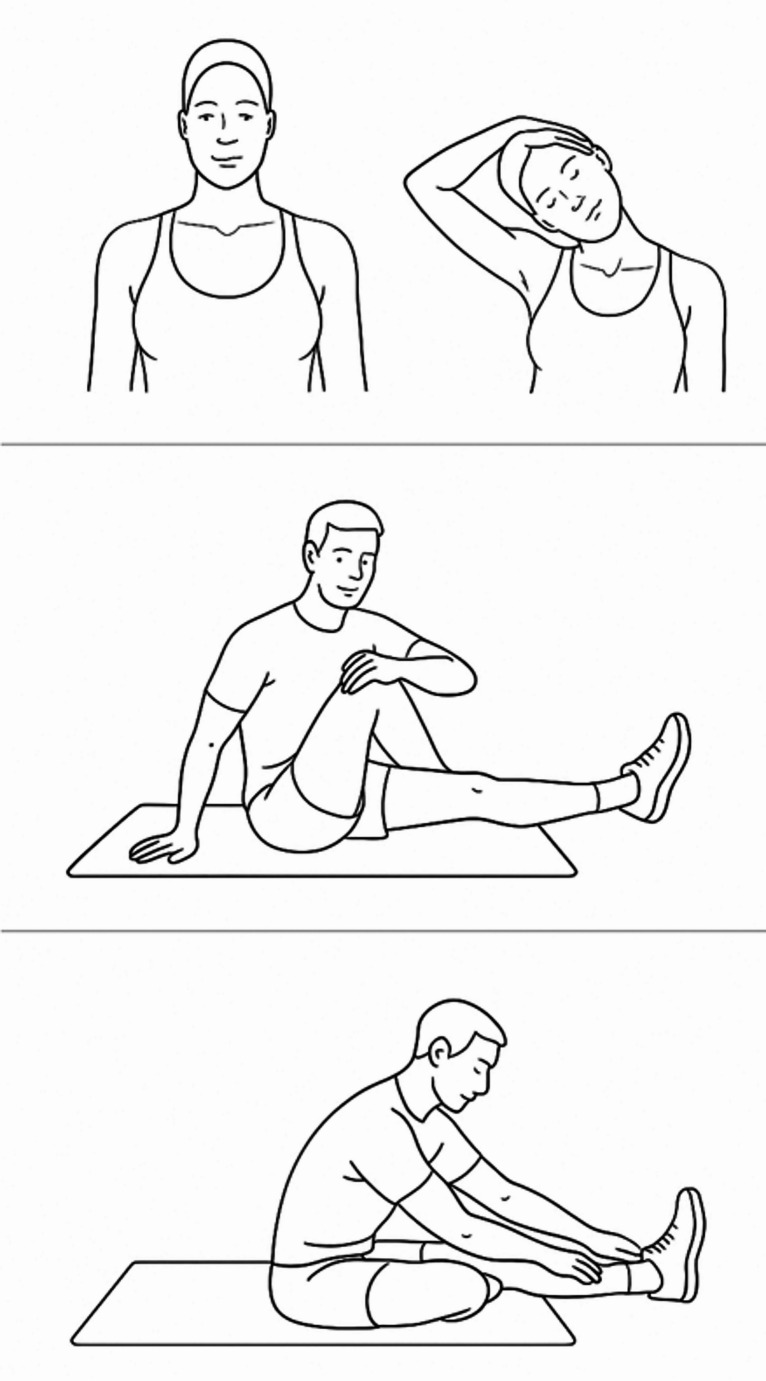
Illustration of the stretching exercises (The figure was generated using ChatGPT based on an abbreviated version of the exercise descriptions as the prompt)

The intervention comprised six weeks of daily static stretching exercises (six minutes a day) in accordance with the recommendation of the American College of Sports Medicine [23]. Following baseline assessments, each participant received individual instruction in the stretching protocol from a physiotherapist (MPS). The self-administered intervention included two 30-second bouts of bilateral static stretches targeting the knee flexors, hip abductors, and shoulder elevators (Fig. 1). Stretches were held at the point of a stretching sensation, with at least 30 s of rest between bouts. The sequence of exercises was flexible, and the composition allowed for minor individual adjustments while remaining consistent across participants. The intervention was reported in accordance with the Template for Intervention Description and Replication (TIDieR) Checklist [24] (Table 1). Adherence was facilitated through a mHealth app (My Physiotherapist, DigiFys, Viborg, Denmark). Participants logged exercise dates, accessed video demonstrations, and communicated via text with the principal investigator (MPS). MPS contacted each participant weekly throughout the trial via the mHealth app. Participants were encouraged to maintain their usual daily routines and asked not to alter their current pharmacological treatments or begin new physical exercise regimens during the study.

**Table 1:** Intervention delivery described using the TIDieR guidelines

	**BRIEF NAME**	
**1.**	Provide the name or a phrase that describes the intervention	Six weeks of app-supported static stretching of the knee flexors, hip abductors and shoulder elevators
	**WHY**	
**2.**	Describe any rationale, theory, or goal of the elements essential to the intervention	Previous studies have found that regular stretching exercises have positive effects on pain, physical and mental functioning, depression, and quality of life in adults with fibromyalgia
	**WHAT**	
**3.**	Materials: Describe any physical or informational materials used in the intervention, including those provided to participants or used in intervention delivery or in training of intervention providers. Provide information on where the materials can be accessed (e.g. online appendix, URL)	A mHealth app (My Physiotherapist) supports adherence to the home-based stretching program. The app also supports video instructions on the stretching procedures
**4.**	Procedures: Describe each of the methods, activities, and/or processes used in the intervention, including any enabling or support activities	The intervention includes self-administered static stretching of the knee flexors, hip abductors and shoulder elevators. Knee flexors: Participants are to perform static stretching of the knee flexors in a seated position. The participant sits upright on the floor with one leg straight. The sole of the other foot is placed on the inside of the outstretched leg. The participant leans slightly forward and tries to touch their toes while maintaining full knee extension Hip abductors: Participants are to perform static stretching of the hip abductors, seated on the floor with both legs extended out in front. The participant bends the left leg at the knee until it touches the chest. The back is kept straight, with the left foot crossed over the right leg, while both hands hug the left knee to the chest. The participant then twists the body toward the left knee. The participant can then pull the left knee to increase the stretch Shoulder elevators: Participants are to perform static stretching of the shoulder elevators in a seated position. The participant sits upright. The participant performs a slight cervical retraction followed by a cervical lateral flexion towards the opposite shoulder The participants are requested to log the daily stretching exercises using the mHealth app. The principal investigator (MPS) will contact each study participant weekly throughout the trial via the mHealth app to monitor adverse responses to the intervention and provide real-time feedback
	**WHO PROVIDED**	
**5.**	For each category of intervention provider (e.g. psychologist, nursing assistant), describe their expertise, background and any specific training given	A physiotherapist (MPS) provides intervention instructions
	**HOW**	
**6.**	Describe the modes of delivery (e.g. face-to-face or by some other mechanism, such as internet or telephone) of the intervention and whether it was provided individually or in a group	Intervention instructions are delivered face-to-face by a physiotherapist (MPS) and are bolstered by video instructions on the mHealth app. The app supports direct message contact between the participant and the principal investigator for Q&A during the study
	**WHERE**	
**7.**	Describe the type(s) of location(s) where the intervention occurred, including any necessary infrastructure or relevant features	Intervention instructions are performed at the research laboratory at the University College of Northern Denmark The stretching intervention is carried out in the participant's home. No infrastructure or relevant features are necessary to carry out the stretching intervention
	**WHEN and HOW MUCH**	
**8.**	Describe the number of times the intervention was delivered and over what period of time, including the number of sessions, their schedule, and their duration, intensity or dose	The self-administered stretching program consists of two bouts of 30-second bilateral static stretches of the knee flexors, the hip abductors and the shoulder elevators, seven days a week for six weeks. The participants are instructed to apply tension to the point of a stretching sensation, holding the stretch for 30 seconds with a minimum of 30 seconds of rest between bouts

### Outcomes

The primary outcome was the participants’ experiences participating in the home-based intervention. We therefore conducted semi-structured focus group interviews to gain insight into the participants’ experience and acceptance of the intervention. Microsoft Teams was selected as the platform for conducting the focus group interviews online to minimise the risk of transportation issues affecting attendance, given the geographical distances in the North Denmark Region. The online focus group interviews were chaired by the same experienced focus group facilitator who had no prior relation to the study (LLL). The interview guide was developed following the framework proposed by Kallio et al. [25]. The interview guide was developed based on current knowledge, consisted of two levels of questions, main themes and follow-up questions and was pilot-tested via expert assessment. The guide contained questions about the participants’ experiences with the intervention and their self-perception of the impact on physical and mental health. The guide drew, in part, on the theoretical framework outlined by Sekhon et al. [26]. It also included questions about the participant’s experiences with the exercise instructions, outcome measurements, and participant information material (supplemental material).

### Secondary outcome measures

Secondary outcome measures were collected at baseline and following the six-week intervention. MPS collected all secondary outcome measures.

#### The impact of FM

The participant’s self-rated overall severity of FM symptoms was assessed using the Danish version of the Revised Fibromyalgia Impact Questionnaire (FIQ-R) [27]. The FIQ-R is the most used instrument to gauge the severity and to identify constellations of symptoms [28]. It consists of 21 questions (items) representing three domains (function, overall severity, and symptoms). Each item is standardised on a scale ranging from 0 to 10, with lower scores indicating less negative impact. The FIQ-R total score ranges from 0 to 100, with higher scores indicating a more significant impact of symptoms [29]. A change in the FIQ-R total score of 14% is considered a minimal clinically important difference (MCID) [30].

#### Health-related quality of life (HRQL)

HRQL was assessed using the Danish version of the Short Form-36 (SF-36) questionnaire [31]. The SF-36 comprises eight health categories, yielding an overall SF-36 score, with higher scores indicating better health outcomes. The categories can be grouped into two second-order summary factors, physical health scores (PCS) or mental health scores (MCS), that are the basis of summary physical and mental health measures. For PCS and MCS, the estimated ranges of a minimal clinically important difference (MCID) are from 2.62 to 4.69 for PCS and 4.46 to 6.79 for MCS [32, 33].

#### Self-reported physical activity

The participants’ perceived physical activity levels were assessed using the short form of the International Physical Activity Questionnaire (IPAQ) [34]. The IPAQ measures time spent in vigorous activity, moderate-intensity activity, walking, and sitting. Results are presented as a continuous variable, expressed in metabolic equivalents (METs) multiplied by minutes over the past week (METs × min/week).

#### Pain sensitivity

Pain sensitivity was expressed using pressure pain thresholds (PPTs), assessed with a handheld digital algometer (Algometer Type 2, SBMEDIC, Hörby, Sweden; 1 cm² probe). Pressure was applied perpendicularly at a rate of 30 kPa/s. Participants pressed a stop button the first time the sensation of pressure was perceived as pain, and the corresponding pressure value was recorded as the PPT. Assessments were conducted at two sites: the right tibialis anterior (1/3 along the line between the fibula tip and medial malleolus) and the left upper trapezius (10 cm from the acromion, aligned with C7). PPTs were measured alternately, three times per site, with 20-second intervals to prevent pain summation. Mean values were used for analysis. Prior to testing, participants were familiarised with the algometer using the left rectus femoris, a non-outcome site.

#### Range of motion

Passive knee extension range of motion was measured using a Biodex System 4 Pro isokinetic dynamometer (Biodex Medical Systems, Shirley, NY, USA). Participants were seated with their hips at 100° and knees at 90° flexion, a position that ensured that tension was placed primarily on the muscle-tendon unit of the knee flexors to prevent hyperextension of the knee during testing [35]. The dynamometer passively extended the knee at 5°/s [36]. Participants pressed a stop button at the transition from stretch to the initial sensation of pain.

All testing was completed at the musculoskeletal laboratory at the University College of Northern Denmark. Stretching exercises are considered safe [37]; hence, no adverse events were expected; possible harms (e.g. soreness or injuries) were monitored using baseline values.

### Sample size

We did not aim for statistical power to be able to identify an effect on the FIQ-R total score or HRQL, but recruited 12 participants to ensure that a sufficient number completed the follow-up to allow us to interpret the qualitative data meaningfully. Generally, a sample size of 12 participants is considered a rule of thumb for pilot and feasibility studies [38].

### Qualitative analysis

The qualitative analysis was based on exploring the participants’ experiences using an inductive approach following the six-step framework for thematic analysis, as described by Braun and Clarke [39]. The interviews were transcribed verbatim, anonymised and inductively coded by MPS using the NVivo 14 software. The codes were then iteratively grouped into themes and sub-themes. Two researchers (MPS & LLL) discussed and reviewed the initial codes, themes and sub-themes and moved back and forth through the data until a consensus was reached to ensure coherence and reflexivity. AR then reviewed the themes and sub-themes. Quotes from the participants were extracted from the data to highlight the emerging themes and sub-themes and translated into English. A narrative analysis was then performed, including an interpretation of the findings and supporting quotes. The qualitative results are reported according to the COnsolidated criteria for Reporting Qualitative Research (COREQ) checklist [40].

### Patient involvement

The main research question was based on prior studies. On September 21, 2023, the study protocol was reviewed by Aalborg University’s Centre for General Practice user panel, consisting of 20 representatives from various patient associations in the North Denmark Region. They provided feedback on the research protocol, participant information, and the intervention burden.

### Statistical analysis

The data were analysed with descriptive and inferential statistics using SPSS 29 (*SPSS Inc.*,* Chicago*,* IL*,* USA*). Continuous variables are presented with mean ± SD. Variables were tested for normality using a visual inspection of histograms and Q-Q plots and a test of deviation from normality (Shapiro-Wilk test). Wilcoxon signed-rank sum tests were used to examine potential differences in pre- and post-test measures. For subjects not assessed post-intervention, intention-to-treat analysis was used with the baseline evaluation carried forward (missing data imputation). An alpha level of 0.05 was defined for the statistical significance of all tests.

## Results

### Enrollment

**Fig. 2:**
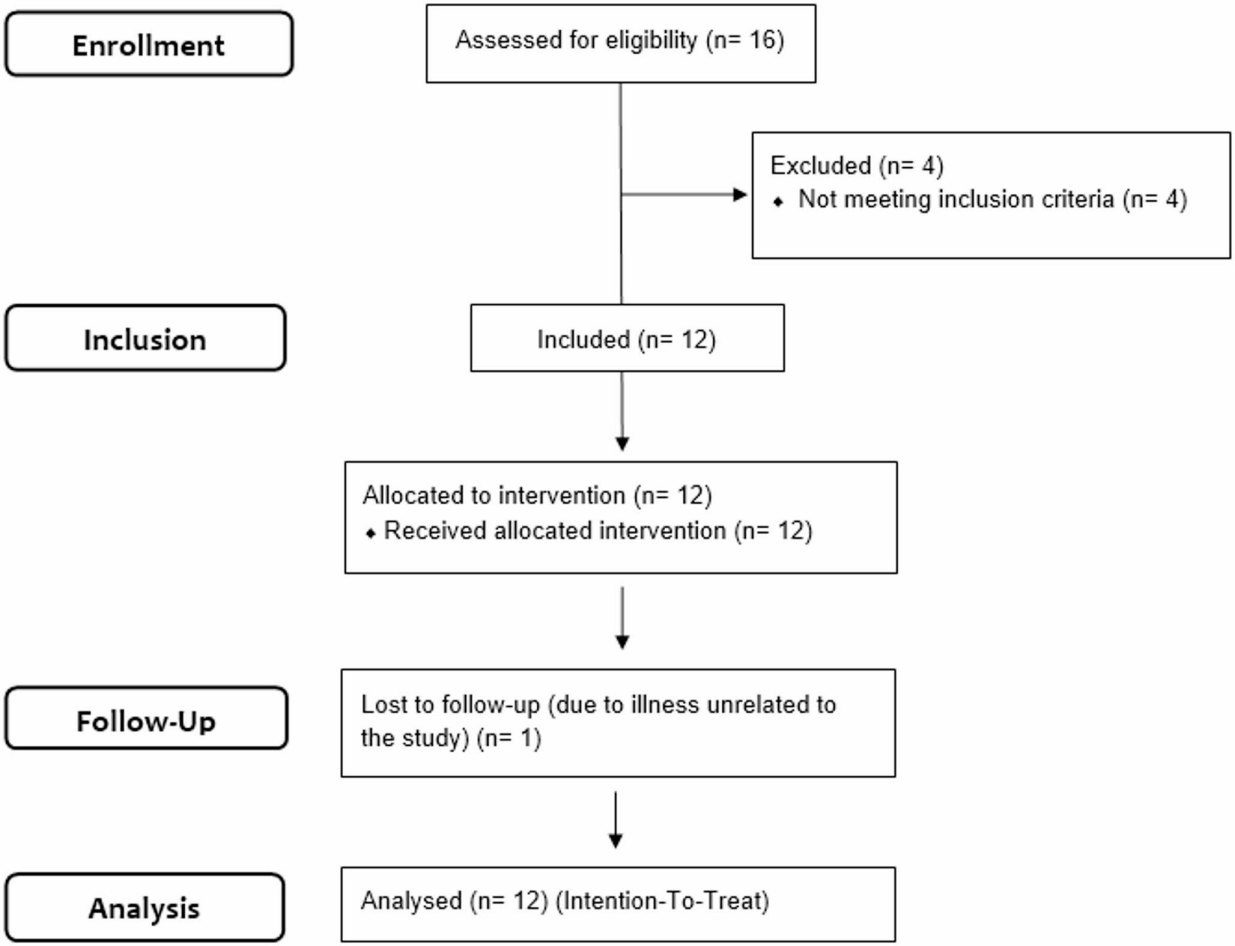
Participant flow diagram

Participants were enrolled in order of initial contact. Twelve participants (*n* = 12 female) were recruited over 43 days. Figure 2 shows a diagram of the participant flow. Nine participants were recruited via posts from the Danish Fibromyalgia & Pain Association, and three were recruited via social media. Following participant enrollment, an additional 10 patients responded to the recruitment posts over the subsequent two weeks.

One participant withdrew from the study due to an illness unrelated to the study. Table 2 shows the demographic and clinical characteristics of the participants.

**Table 2:** Demographic and clinical characteristics

Characteristic	
N	12
Age, mean (SD) years	46.2 (8.8)
Height, mean (SD) meters	1.69 (5.4)
Body mass index, mean (SD)	28.8 (5.0)
Time since diagnosis, mean (SD) years.	4.2 (3.6)
Time since the first onset of symptoms, mean (SD) years.	14.3 (7.5)

The total time spent on weekly follow-ups with the 12 participants via the app amounted to 2.5 h over the study period, equating to less than 20 min per participant across the six weeks. Log data revealed that the majority of contacts during the study were initiated by the principal investigator (MPS).

### Qualitative results

Two online focus-group interviews were conducted in May 2024. Eight of the 11 participants were able to participate in the interviews. One participant declined the invitation, one was unavailable due to work engagements, and one fell ill on the interview day. Each online focus group interview involved four respondents and lasted approximately 60 min.

**Fig. 3:**
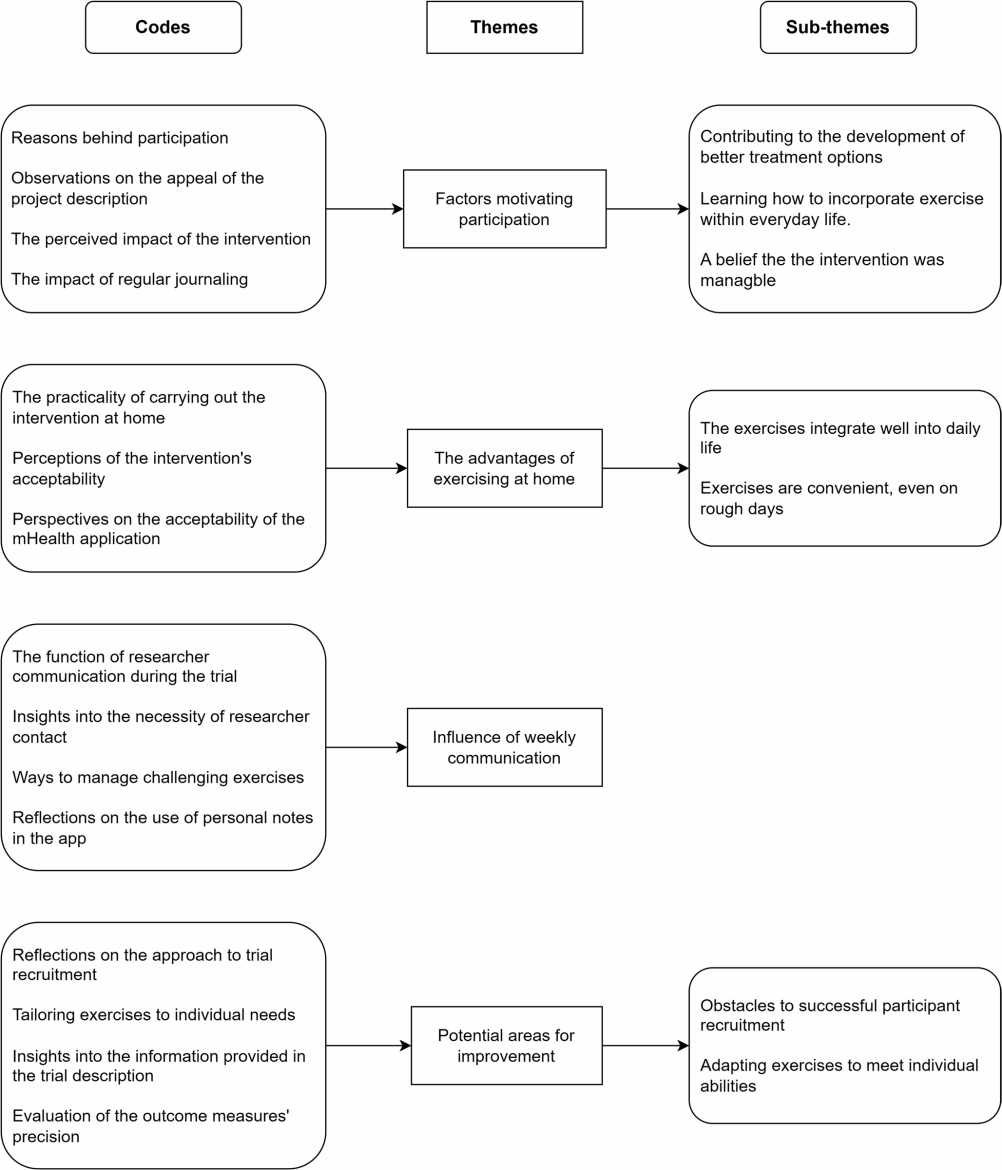
Codes, major themes and subthemes identified through the inductive analysis

Based on the qualitative interviews, we identified four major themes. Three themes emerged relating to the acceptability of the intervention, while one theme pertained to the research processes. The major themes were: (1) Factors motivating participation, (2) The advantages of exercising at home, (3) Influence of weekly communication and (4) Potential areas for improvement. Three of the four major themes were divided into subthemes. Codes, major themes and subthemes are summarised in Fig. 3.

#### Theme 1: factors motivating participation

Factors motivating participation describe a theme that catches the participants´ perspectives on what drove their incentive to participate in the study. It comprises three sub-themes, each addressing the variability in factors that encouraged participation.

1. Contributing to the development of better treatment options.

The participants expressed that they often feel overlooked by the traditional healthcare system. This manifested as both challenges in accessing effective treatments and a perceived lack of research on developing new treatments for FM. Consequently, they feel obligated to contribute to developing new therapies when possible.

*“No one’s helping us*,* so I figured I’d donate my body for this; it couldn’t possibly hurt*,* and I might learn something new.” (pt. 4)*.

Furthermore, the participants also pointed out a profound sense of shared responsibility, suggesting that individuals with the means and resources to participate in research should do so for the benefit of others.

*“I believe you should do everything you can to help others with fibromyalgia in the future.” (pt. 5)*.

2. Learning how to incorporate exercise into everyday life.

The participant expressed that a motive for participation was to learn more about incorporating exercise into their daily life. Many previously led active lives, but following their diagnosis, their tolerance for physical activity had declined to a point where they found it increasingly difficult to cope with basic activities of daily living, much less implement new physical activities.

*“I know that staying active is good for me. I used to be very active*,* but since my diagnosis*,* I’ve had trouble understanding how I respond to physical activity. I often have setbacks when I start something new.” (pt. 4)*.

Moreover, participants expressed confusion about how to increase their physical activity levels. They wanted to integrate exercise into their daily routines while managing family and work responsibilities, but struggled to find the mental and physical energy to do so.

*“I feel a bit lost. I used to be active*,* but I don’t have the energy or mental space for it. I want to find a compromise where I can exercise a little and still manage my work and family life*,* but it’s hard with this diagnosis.” (pt. 1)*.

3. A belief that the intervention was manageable.

The participants shared that the description of the home-based intervention was an encouraging factor for participation. Many had a negative perception of physical exercise, attributing it to increased FM symptoms. However, stretching exercises carried a more positive connotation. Accordingly, the participants felt that the intervention was something they could manage.

*“I associate exercise with something unpleasant that makes me very tired*,* but this (stretching intervention) felt easier to manage.” (pt. 2)*.

#### Theme 2: the advantages of exercising at home

The advantages of exercising at home describe a theme that emerged from the participants´ perspectives on how the home-based intervention fitted into their daily lives. It comprises two sub-themes, each addressing different aspects of how the participants experienced blending the exercises into daily life.

1. The exercises integrate well into daily life.

The participants shared positive experiences with fitting the exercises into their daily schedule and incorporating them into their daily routines despite balancing work and family responsibilities.

*“I fit the exercises into my evenings because the mornings are all about getting the kids ready for school and myself ready for work.” (pt. 3)*.

One participant even described how the exercises became fun and engaging for her kids.

*“The kids enjoyed it*,* so we often did the exercises together*,* me and the little girls*,* whenever it fit into our day.”(pt. 8)*.

2. Exercises are convenient, even on rough days.

The participants highlighted that the exercises were convenient and gentle. This meant they could perform the exercises even when their symptoms were most severe without exacerbating them.

*“I get so tired these days that it hurts deep in my bones*,* but even though I’m completely drained and it feels like everything is a mess*,* I can still do the exercises.”(pt. 3)*.

#### Theme 3: influence of weekly communication

The influence of weekly communication describes a theme that emerged from the participants´ perspectives on the influence of weekly communication with the principal investigator via the mHealth application. Some participants experienced the need to modify specific exercises during the trial, but found it easy and convenient to use the app to seek advice on making individual adjustments to address the challenges.

*“At one point*,* I had an issue with one of the exercises*,* but it was easy to just write (to the principal investigator) and ask for advice.” (pt. 5)*.

Most participants did not feel the need to communicate with the principal investigator during the trial. While they responded to the weekly messages, they did not initiate contact themselves. Still, they found comfort in knowing that assistance and guidance were available.

*“It was comforting to know that if you had questions or needed help*,* someone was always there for you.” (pt. 8)*.

The participants expressed that while they did not view the monitoring negatively, the weekly communication helped them stay consistent with the exercises.

*“It’s comforting to know he (the principal investigator) is there. It is reassuring to know that you are not just doing the exercises for no reason*,* but that there is someone on the other end with whom you are helping and doing something together.” (pt. 5)*.

##### Theme 4: potential areas for improvement

Potential areas for improvement describe a theme that emerged from the participants´ perspectives on the study design and intervention, offering ways to enhance the study’s impression. It comprises two sub-themes, addressing different aspects of factors related to areas for improvement.

1. Obstacles to successful participant recruitment.

A key participant observation was the identification of potential obstacles to recruitment for this kind of intervention study. The participants expressed that they might be among the better-functioning patients, as not all people with FM share their energy level or mental fortitude. They explained that for many, the thought of doing exercises daily, even if for just six minutes, could present a barrier to participation:

*“Not everyone shares our level of energy. For some*,* doing the exercises daily*,* even for six minutes*,* could simply*,* on the face of it*,* be an overwhelming challenge.”(pt. 4)*.

Furthermore, the participant also voiced concerns about how best to promote the study and establish contact with potential participants. They revealed that they have private social media groups where FM patients can exchange experiences and build connections, but these groups are accessible only with permission from current members.

*“We’re not registered anywhere*,* so it’s hard to get in touch with us. We have private groups on Facebook*,* but you need permission to join them.” (pt. 2)*.

2. Adapting exercises to meet individual abilities.

The participants highlighted that although stretching exercises generally have a positive connotation, it might be necessary to clearly state that the exercises can be adjusted to individual needs to attract those with moderate to severe limitations in functional capacity.

*“It’s important to emphasise that the exercises can be adjusted to accommodate different needs.”(pt. 4)*.

### Quantitative outcomes

Since one participant was not assessed post-intervention, an intention-to-treat analysis was used with the baseline evaluation for FIQ-R, SF-36, pressure pain thresholds and range of motion measures carried forward. Mean values ± SD and percentage differences for FIQ-R, SF-36, pressure pain thresholds and range of motion are presented in Table 3.

**Table 3:** Absolute values (means (SD) and percentage differences) for secondary outcomes

	Baseline	Post-stretch	% difference
FIQ-R			
Total score	56.5 ± 16.6	44.5 ± 14.5	21.2
Physical Function	14.3 ± 6.8	11.8 ± 5.3	17.5
Symptoms	29.6 ± 7.5	22.8 ± 6.8	22.8
Overall	12,. ± 4.7	9.5 ± 4.8	25.0
Pain	5.8 2.6	4.6 ± 2.8	20.3
Stiffness	5.8 ± 2.6	4.4 ± 2.6	23.2
SF-36			
MCS	32.2 ± 5.9	36.0 ± 5.0	11.8
PCS	36.3 ± 6.3	41.7 ± 7.7	15.0
PPT—Tibialis Anterior	125.9 ± 32.2	108.4 ± 14.0	-13
PPT—Trapezius	129.7 ± 36.3	126.9 ± 42.8	-2
ROM	157.9 ± 20.5	159.8 ± 23.2	1,2

#### FIQ-R

There were clinically relevant improvements in the FIQ-R Total score (mean change = 12 ± 8.3; *p* = 0.003), function (mean change = 2.5 ± 3.4; *p* = 0.036), symptoms (mean change = 6.8 ± 4.6; *p* = 0.005), overall impact (mean change = 3.2 ± 3.6; *p* = 0.015), and stiffness (mean change = 1.3 ± 2.1; *p* = 0.041). There were, however, no clinically relevant differences in pain (mean change = 1.2 ± 1.9; *p* = 0.065).

### SF-36

There were clinically relevant improvements in PCS (mean change = 3.8 ± 5.3; *p* = 0.026) and MCS (mean change = 5.4 ± 6.1; *p* = 0.013).

#### Pressure pain thresholds and range of motion

There were no clinically relevant changes in flexibility (mean change = 1.8^°^±14.9; *p* = 0.515) or pain sensitivity (mean change > 2.8 ± 23.6; *p* > 0.130).

#### Self-perceived physical activity

There was a significant increase in the participants’ self-perceived physical activity levels (MET score) (*p* = 0.021).

#### Adherence to the intervention

For self-reported adherence, data from the 11 participants who completed the intervention and were assessed post-intervention are reported. The self-reported adherence rate was 91% (± 6.9%).

## Discussion

The present study investigated the feasibility and acceptability of a six-week home-based stretching programme supervised via a mHealth application for people with FM. The findings demonstrated that the intervention is feasible in terms of recruitment rate, retention at follow-up, quality of life, physical and mental function, and adherence. The intervention was found acceptable by the participants, with some barriers relating to successful participant recruitment and adaptability of the exercises to meet individual needs and physical conditions that needed to be amended before proceeding to the RCT [21].

Patients with FM are often intolerant of physical activity [41]. Many experience exercise-induced worsening of symptoms when initiating physical exercise programs [42]. Consequently, although the stretching exercises were low-dose, it was essential to examine how the participants reacted to the daily routines. The results add to current knowledge, showing that the daily stretching exercises were well tolerated, with no adverse events or harms reported. Notably, findings from the qualitative data showed that the stretching exercises could be performed even during periods of severe symptoms without exacerbating them. This is encouraging and supports further examination of the clinical efficacy of home-based stretching exercises.

To mitigate the risk of reduced adherence, participants were asked to log the dates they performed the stretching exercises, and weekly text-based interactions were facilitated through the mHealth app throughout the trial. While we recognised that exercise logging might make participants feel scrutinised or pressured, all feedback was positive. Participants reported that the logging feature supported adherence without making them feel monitored or judged. They appreciated the reassurance of having access to guidance when needed. These findings align with previous observations suggesting that app-based supervision in home-based exercise interventions can enhance motivation and improve adherence [43].

The resources required for the weekly follow-ups were initially uncertain, and it was unclear to what extent participants would initiate contact via the app. However, as the total follow-up time amounted to less than 20 min per participant over the six weeks, the approach appears suitable for patients with FM and feasible for implementation.

Findings from the qualitative data indicated the need for intervention revisions before initiating the randomised controlled trial (RCT). (1) To reduce the risk of potential barriers to recruitment, we decided to revise the project description to highlight that the exercises are gentle, can be performed everywhere, and can be easily modified to fit the needs and physical limitations of those who participate. (2) We also developed new video tutorials and exercise descriptions on the mHealth app, explaining how and when to modify each exercise [21].

The feasibility study was not powered to study the intervention’s effect size, which prevents the generalisability of the results. Yet, the quantitative results showed clinically relevant improvements in quality of life and physical and mental health scores. This aligns with findings in the most recent systematic reviews indicating potential improvements in pain, health-related quality of life and physical and mental functioning in people with FM following stretching exercises [14, 17].

In this study, the intervention period was six weeks. Consequently, the duration of the intervention was shorter than in previous related studies [44–46]. However, based on emerging research, we believed that six weeks would be sufficient to demonstrate potential treatment effects [47]. It is possible that this approach only examines the short-term effect. However, existing evidence indicates that stretching exercises may lead to both short- and long-term clinically significant improvements in quality of life [45, 46].

The fact that the exercises were the same for all participants may be viewed as a limitation in the delivery of exercise. However, current evidence shows that the efficacy of stretching exercises is non-local and affects non-stretched muscles and joints [48]. Also, current evidence indicates that stretching exercises produce a widespread analgesic effect, likely mediated by endogenous mechanisms modulating somatosensory input [19–22]. We therefore chose to include three simple exercises designed to be accessible and manageable for all participants, regardless of their functional capacities. Even though the sequence of exercises was flexible, and the composition allowed for minor individual adjustments, several participants noted that the ability to further adapt the exercises to individual needs and resources would be a valuable improvement to the RCT protocol.

According to existing evidence, only 21% of patients with FM referred to specialist rehabilitation in Denmark are part of the workforce [49, 50]. To increase the likelihood of including participants who were part of the workforce, acknowledging that their barriers to daily exercise may differ from those on long-term sick leave or receiving social welfare payments, we defined the inclusion age range as 18 to 55 years in the present feasibility study.

Based on the results of the present study, we calculated the sample size for the RCT. The sample size was calculated using repeated-measures analysis of variance for a design with a 2-level within-subject factor and a 2-level between-subject factor. The mean baseline FIQ-R total score was set at 56.5, and the covariance matrix for repeated measurements, assumed to be constant across both groups, was set to [276 209/ 209 211]. With an alpha level of 0.05, assuming no change in the score in the control group and a minimal clinically relevant score change of 14% in the intervention group, 24 patients per group are required to achieve a power of 0.9. Additionally, considering a 20% loss to follow-up, the final total sample size needed is 58 (29 in each group) [21].

The findings from this feasibility and acceptability study support proceeding to the randomised controlled trial. The future RCT will provide knowledge of the clinical effects of a home-based stretching program supervised via an mHealth application compared with usual care alone, which can hopefully help reduce the burden of symptoms experienced by people with FM. Additionally, it will be interesting to study if the participants in the RCTcan maintain the home-based exercises in the long term.

### Strengths and limitations

A strength of the study is the involvement of patients in planning the study design and recruitment process. Additionally, integrating both quantitative and qualitative methods provides a detailed and clinically valuable insight into the intervention’s feasibility and potential clinical relevance. The limitations of this study are the basic methodological constraints of a one-armed feasibility design, including limited sample size, the absence of a control group, and the lack of blinding for participants and researchers, which prevent drawing conclusions about the intervention’s clinical efficacy. It is also a limitation that all participants were female, as this reduces the generalisability of the results.

The online focus group interviews included all eight participants involved in the intervention who consented to interviews. Expanding the sample size was not feasible. Additional insights might have emerged with a larger sample. As a result, the small sample size represents a limitation in interpreting the qualitative findings.

## Conclusion

A six-week home-based stretching program, supervised via an mHealth application, proved feasible and acceptable for individuals with FM and showed promising clinical outcomes. Based on insights from this feasibility study, an RCT is planned to evaluate whether the program of home-based stretching exercises provides greater benefits than usual care alone in enhancing quality of life and functional outcomes in patients with FM.

## Data Availability

The datasets used and analysed during the current study are available from the corresponding author upon reasonable request. The interview guide is available as supplemental material.
